# Incidence and Relative Risk of COVID-19 in Adolescents and Youth Compared With Older Adults in 19 US States, Fall 2020

**DOI:** 10.1001/jamanetworkopen.2022.22126

**Published:** 2022-07-15

**Authors:** Moshe Schneiderman, Barbara Rumain, Leon Kaganovskiy, Allan Geliebter

**Affiliations:** 1 SUNY Downstate College of Medicine, Brooklyn, New York; 2 Department of Pediatrics, New York Medical College, Valhalla, New York; 3 Department of Psychology, Touro University, New York, New York; 4 Department of Mathematics, Touro University, New York, New York; 5 Department of Psychiatry, Icahn School of Medicine at Mount Sinai, New York, New York

## Abstract

**Importance:**

Adolescents have been thought to be low in susceptibility to COVID-19 compared with older adults. Data regarding incidence and risk of COVID-19 are needed to convey risk of infection and inform prevention messaging, especially because US states such as Florida are recommending against vaccinating individuals ages 5 to 17 years and because more infections among adolescents could signal potentially higher incidence of long COVID.

**Objective:**

To compare incidence rates and relative risk of infection among US adolescents and youth with those of older adults for wild-type SARS-CoV-2.

**Design, Setting, and Participants:**

This cross-sectional study included persons living in 19 US states that experienced surges from the start of the pandemic through fall 2020. Participants were all individuals reported as cases on state health department websites for the age groups and states studied. Age groups included adolescents (ages 10 to 19 years), youth (ages 15 to 24 years or 18 to 24 years), adolescents and youth combined (ages 10 to 24 years), and older adults (either age 60 years or 65 years and older), with age thresholds dependent on individual state data. Data were analyzed between June 2021 and January 2022.

**Main Outcomes and Measures:**

Incidence rates for the wild-type SARS-CoV-2 strain; the relative risk of infection in adolescents and youth compared with older adults, based on the incidence rate ratio (IRR).

**Results:**

In 16 of 19 states, the IRR of COVID-19 infection in adolescents and youth was significantly greater than in older adults. For example, in Florida, the incidence rate in adolescents and youth was 0.055 compared with 0.028 in older adults—adolescents and youth had 1.94 times the risk of contracting COVID-19 compared with older adults (IRR, 1.94; 95% CI, 1.92-1.95).

**Conclusions and Relevance:**

Results from this cross-sectional study with US data were contrary to studies from Asia and Europe indicating lower susceptibility of adolescents than older adults. Our findings with the wild-type strain were consistent with findings reported in the UK for the Delta variant and underscored that even with the wild-type lineage, incidence among adolescents and youth exceeded that in older adults.

## Key Points

**Question:**

How do incidence and relative risk of contracting COVID-19 from the original wild-type SARS-CoV-2 strain in adolescents and youth compare with that in older adults in the US?

**Findings:**

Results of this cross-sectional study using state health department data from the start of the pandemic through fall 2020 indicate that, in 16 of the 19 states examined, the incidence rate and relative risk of COVID-19 infection from wild-type SARS-CoV-2 were significantly greater in adolescents and youth than in older adults. For example, in Florida, the incidence rate in adolescents and youth was 0.055 compared with 0.028 in older adults—adolescents and youth had 1.94 times the risk of contracting COVID-19 compared with older adults.

**Meaning:**

These results suggest that, contrary to reports from Europe and Asia, infection rates and relative risk among US adolescents and youth exceeded that in older adults from the start of the COVID-19 pandemic through fall 2020, before vaccines were available.

## Teaser

This cross-sectional study compares rates of COVID-19 incidence between adolescents and youth with older adults in 19 US states in 2020.

## Introduction

The susceptibility of adolescents (ie, ages 10 to 19 years) and youth (ages 15 to 24 years) to COVID-19 has been a matter of controversy. In early studies conducted in China, Dong et al,^1^ Lu et al,^2^ and Bi et al^3^ reported adolescents were quite susceptible, with Bi et al^3^ reporting similar infection rates across all age groups. However, Zhang et al^4^ concluded that older adults were most susceptible, those in the first half of adolescence least susceptible, and youth intermediate in susceptibility. Geliebter, Rumain, and Schneiderman^5^ attempted to replicate Zhang et al’s analyses but obtained results in line with Bi et al, indicating similar infection rates across age groups. Data from Europe also indicated adolescents were less susceptible than adults (Kuchar et al^6^ in Warsaw; de Lusignan et al^7^ in England). Furthermore, Viner et al,^8^ after a meta-analysis of 32 studies, concluded that “children and adolescents younger than 20 years had 44% lower odds of secondary infection with SARS-CoV-2 as compared with adults 20 years and older.” Subsequently, a mathematical model by Eggo and colleagues,^9^ based on data from China, Italy, Japan, Singapore, Canada, and South Korea, estimated the susceptibility of adolescents ages 10 to 19 years as less than half that of adults ages 60 years or older. However, no US data were included in their model.

In a 2022 study in* JAMA Pediatrics*, Dawood et al^10^ examined the incidence rates of SARS-CoV-2 in households in New York and Utah from September 2020 to April 2021. They found that children and adolescents had similar rates of infection as adults. The study did not account for the mass vaccination efforts during this time period: on December 14, 2020, the first COVID-19 vaccine doses were administered, and by April 21, 2021, the milestone of 200 million doses was achieved,^11^ which would decrease the incidence rate in adults. However, individuals aged 16 years first became eligible for the vaccine on April 19, 2021,^12^ and adolescents ages 12 to 15 years on May 10, 2021.^13^ Their timeline also conflated various strains of SARS-CoV-2: in September 2020, the wild type was circulating, but by April 24, 2021, mainly variants were circulating (eFigure 1 in the Supplement).^14^

The purpose of this study was to investigate the incidence of COVID-19 due to wild-type SARS-CoV-2 in adolescents (ages 10 to 19 years) and youth (15 to 24 years) compared with older adults. We selected these 2 age brackets because children and adolescents are generally considered the least susceptible to COVID-19, and older adults the most vulnerable (eg, in Eggo’s model). We also included youth because the age bracket overlaps with adolescence. We examined the incidence rate, the number of new cases of COVID-19 as a proportion of the number of people in each age group, from the start of the pandemic through fall 2020, the timeframe during which the wild type was circulating and when vaccines were still unavailable.

## Methods

This study was exempt from institutional review board (New York Medical College) and informed consent requirements because it used deidentified data. It followed the Strengthening the Reporting of Observational Studies in Epidemiology (STROBE) reporting guideline.

We focused on 19 states that met 2 criteria. The first was that the state experienced a surge during fall 2020, defined as an increase of at least 75% from a plateau 2 to 3 months earlier that lasted at least 1 month (eAppendices 1 and 2 in the Supplement).^15^ For example, the case data for Colorado are from October 22, 2020, when there was a surge. On that day, the 7-day average number of daily new cases statewide was 1171. Two months earlier, on August 22, the 7-day daily average was 296 cases, for a 300% increase. From June to August 21, the 7-day daily average had plateaued at 200 to 500 cases per day.

By *plateau*, we mean there is minimal variation in the 7-day average of daily number of new cases in a given period of time that is at least 1 month in duration. This can be measured using the variance of the 7-day averages over the given time interval. For example, if we compare the variances of the 7-day averages in daily new cases in Colorado for the month of August 2020 to that of November 2020, we observe that the August variance is significantly lower than the November variance (Levene test for equality of variances, *P* < .001).

The second criterion for inclusion were that states had available pediatric data that were tabulated within distinct age brackets. California lumped all child data 0 to 17 years of age together and could not be included, as children under age 10 years were excluded in our study. Massachusetts lumped children ages 0 to 19 years together, as did Arizona. We therefore considered the following 19 states: Alabama, Alaska, Colorado, Florida, Michigan, Minnesota, Missouri, Montana, Nebraska, Nevada, New Mexico, North Dakota, Oklahoma, Oregon, Pennsylvania, Rhode Island, South Carolina, Tennessee, and Wisconsin. We accessed online COVID-19 case data from state health department websites when there was a surge, and tables for state population data by age group. Case data were downloaded from October 22 through December 5, 2020, at the time each state experienced a spike in cases. Websites and relevant figures and tables are shown in eAppendix 3 in the Supplement. Depending on how the data were tabulated, the case data for the 19 states were either for adolescents (ages 10 to 19 years), youth (15 to 24 years), or adolescents and youth combined.

### Adolescent, Youth, and Combined Population Data

In the following 13 states, data were tabulated by decade, and we examined individuals aged 10 to 19 years: Alaska, Colorado, New Mexico, Michigan, Wisconsin, Montana, South Dakota, North Dakota, Oregon, Nevada, and Pennsylvania. Tennessee had a similar age bracket of 11 to 20 years. Florida, Oklahoma, Rhode Island and Alabama, provided data on youth. The age brackets for Florida and Oklahoma were 5 to 14 years and 15 to 24 years, and we used only 15 to 24 years for both states. We also used the 15-to-24-year age bracket for Rhode Island. For Alabama, cases were reported for 0 to 4 years, 5 to 17 years, and 18 to 24 years, age bracket demarcations that were unlike the other states; we used the 18-to-24-year age bracket, since it is a subset overlapping our definition of youth. Minnesota and Missouri provided data on adolescents and youth combined (ie, individuals aged 10 to 24 years).

### Statistical Analysis

We calculated 2 measures: incidence rate and incidence rate ratio (IRR). Incidence refers to the number of individuals who developed COVID-19 from the start of the pandemic through fall 2020. The incidence rate for a given age group in a given state is the number of cases in the age group in the state since the start of the pandemic through fall 2020 divided by the number of individuals in the population living in the state who are in the given age group (eAppendix 4 in the Supplement).

Although our data from fall 2020 were over differing durations (for example the end point for Colorado was October 22, whereas for South Carolina it was December 5), this is inconsequential because the comparisons were not across states but between age groups within a given state, and the time period within a state would be the same for adolescents plus youth and for older adults.

For each age group in each state, we converted each proportion to an incidence rate.^16^ We then calculated the IRR using the formula: (incidence rate for adolescents or youth) / (incidence rate for older adults). We computed the IRR and associated 95% CIs of contracting COVID-19 for adolescents and youth vs for older adults using Mosaic package in R version 1.8.3 (R Project for Statistical Computing). We also performed a sign test, a nonparametric test to compare the numbers in paired groups (adolescents and youth vs older adults) in each of the 19 states; the threshold for significance was 2-sided *P* < .05.

As per the STROBE reporting guidelines, we considered potential sources of bias. Because adults are more often tested for COVID-19 than adolescents, this would introduce a sampling bias: the more testing done, the greater the likelihood of finding cases. Hence, this sampling bias would tend to increase the number of cases reported in older adults. Also, our own personal observations have been that testing centers are reluctant to test adolescents because they think adolescents will experience only mild symptoms, if any. Thus, the numbers of cases reported for adolescents on the state health department websites may be underreporting actual numbers of cases.

## Results

In 16 of the 19 states experiencing surges during fall 2020, the incidence rate of COVID-19 was significantly higher in adolescents and youth than it was in older adults (Table). It was statistically higher because the IRR was significantly greater than 1 in 16 of the 19 states, indicating that adolescents had an increased relative risk of contracting COVID-19 compared with older adults.

**Table.** Incidence and IRRs by Developmental Period and Age Bracket in US States Experiencing Spikes in COVID-19 Cases

State	State data end date^a^	Adolescents, youth, or combined		Older adults		IRR (95% CI)
		Cases/cohort population, No.	Incidence rate	Cases/cohort population, No.	Incidence rate	
**Adolescents (ages 10-19 y) vs older adults (ages ≥60 y)**						
Alaska	October 31	1811/99 499	0.018	2330/142 099	0.016	1.12 (1.04-1.18)
Colorado	October 22	9890/731 951	0.014	15 476/1 199 263	0.013	1.05 (1.02-1.07)
Michigan	November 17	37 393/1 267 877	0.030	92 591/2 393 510	0.039	0.76 (0.75-0.77)^b^
Montana	October 23	3001/137 796	0.022	5668/286 567	0.020	1.10 (1.05-1.15)
Nevada	October 30	8727/391 347	0.022	14 107/683 039	0.021	1.08 (1.05-1.11)
New Mexico	October 23	4513/285 393	0.016	6726/518 073	0.013	1.22 (1.17-1.26)
North Dakota	October 23	4658/97 348	0.048	7143/167 040	0.043	1.12 (1.08-1.16)
Oregon	October 30	4840/504 711	0.010	7136/985 350	0.007	1.33 (1.28-1.37)
Pennsylvania	October 30	18 319/1 568 292	0.012	55 956/3 301 963	0.017	0.69 (0.68-0.70)^b^
South Carolina	December 5	33 151/673 843	0.049	49 368/1 211 555	0.041	1.21 (1.19-1.22)
South Dakota	October 23	4052/117 276	0.035	7569/225 553	0.034	1.03 (0.99-1.07)^c^
Tennessee	November 11	38 925/855 574	0.046	52 404/1 577 807	0.034	1.35 (1.34-1.37)
Wisconsin	October 23	22 857/748 773	0.031	34 286/1 428 853	0.024	1.27 (1.25-1.29)
**Youth (ages 15-24 or 18-24 y) vs older adults (ages ≥60 or ≥65 y)**						
Alabama^d^	November 13	25 413/458 530	0.055	30 186/854 313	0.035	1.57 (1.54-1.59)
Florida^e^	November 12	140 515/2 555 315	0.055	126 647/4 465 169	0.028	1.94 (1.92-1.95)
Oklahoma^e^	October 22	22 499/543 700	0.041	15 853/635 222	0.025	1.66 (1.63-1.69)
Rhode Island^f^	October 24	4696/145 880	0.032	7017/265 058	0.027	1.22 (1.17-1.26)
**Adolescents plus youth (ages 10-24 y) vs Older adults (ages ≥65 y)**						
Minnesota	October 23	32 186/1 081 469	0.030	16 017/921 491	0.017	1.71 (1.68-1.75)
Missouri	October 23	28 634/1 192 555	0.024	19 278/1 033 964	0.019	1.29 (1.27-1.31)

Abbreviation: IRR, incidence rate ratio.

^a^ All data collection dates in 2020.

^b^ Opposite direction from the other states.

^c^ Not significantly different.

^d^ Youth ages 18 to 24 years vs older adults 65 years or older.

^e^ Youth ages 15 to 24 years vs older adults 65 years or older.

^f^ Youth ages 15 to 24 years vs older adults 60 years or older.

### Adolescents vs Older Adults

The incidence rate in adolescents was significantly greater than in older adults in 10 of 13 states (eg, in Tennessee, incidence rate in adolescents, 0.046 vs older adults, 0.034; IRR, 1.35; 95% CI, 1.34-1.37) (Table). For South Dakota, there was no significant difference between adolescents and older adults. For Michigan and Pennsylvania, the pattern was reversed, with the incidence rate in older adults being significantly greater than in adolescents (eg, Michigan: incidence rate in adolescents, 0.030 vs older adults, 0.039; IRR, 0.76; 95% CI, 0.75- 0.77), indicating adolescents had 0.76 times the risk of contracting COVID-19 than older adults (Table).

### Youth vs Older Adults

We found in all 4 states that youth (15 to 24 years of age) had a greater incidence rate of COVID-19 infection than older adults. For example, in Florida, the relative risk of youth 15 to 24 years old contracting COVID-19 was 1.94 that of older adults (95% CI, 1.92-1.95) (Table).

### Adolescents Plus Youth Combined vs Older Adults

In Minnesota and Missouri, the incidence rate of COVID-19 in individuals aged 10 to 24 years was significantly greater than in adults 65 years or older. For example, in Minnesota, the incidence rate in adolescents was 1.71 times that in older adults (95% CI, 1.68-1.75) (Table).

In summary, for the 19 states experiencing surges during Fall 2020, the incidence rate of COVID-19 was significantly higher in adolescents or youth than it was in older adults. In 16 of the 19 states, there was a significant increase; in 2 states, there was a significant decrease; and in 1 state (South Dakota), there was no difference (sign test, *P* < .001).

## Discussion

In 16 of the 19 states experiencing surges of cases during fall 2020, we found the incidence rate of COVID-19 was significantly higher in adolescents and youth than in older adults. Our data were from the original wild-type SARS-CoV-2 strain, as these data were from October to November 2020 (with the exception of South Carolina, which was from December 5, 2020), all before the report of any variants in the US.^17^ Moreover, our findings of lower incidence in older adults cannot be attributed to access to vaccination because in fall 2020 vaccinations were unavailable. Possible reasons for our findings are that adolescents had more contacts than adults^18^; and that older adults, feeling vulnerable, were more likely to adhere to masking and/or social distancing guidelines than adolescents and youth.

Our findings in the 16 US states differed from those of Zhang et al^4^ in China, who found the infection rate in older adults ages 65 years and older exceeded that in adolescents and youth, and from those of Wu et al,^19^ who found that of 44 672 confirmed cases of COVID in China, only 1% were in adolescents ages 10 to 19 years. Our findings also contradicted Eggo’s model, based on data from Asia and Europe for the original wild-type strain, which estimated the susceptibility of individuals aged 10 to 19 years to be half that of older adults.^9^ The reason for the discrepancy could be that these earlier studies were conducted when schools were closed, which reduced contacts by adolescents and youth and therefore limited case volume. Moreover, testing was not readily available early in the pandemic, and adolescents tended to have milder cases that could have been missed without available widespread testing. Indeed, as of April 2, 2020, among 149 082 cases in all age groups for which age was known, only 1.7% of these occurred in children younger than 18 years, and of these, nearly 60% occurred in adolescents 10 to 17 years old.^20^ Hence at that point, adolescents accounted for just 1% of the total cases. By September 15, 2020, a month or 2 before our data were collected, the cases in adolescents ages 10 to 19 years had climbed to 387 000.^21^ And, as of May 19, 2022, the American Academy of Pediatrics reported 13 253 639 total child COVID-19 cases in children younger than 18 years with at least 1032 deaths.^22^

The 3 states that were exceptions could be the result of certain events in these states. The Sturgis Motorcycle Rally could help explain the lack of a difference in South Dakota. Data from South Dakota were from October 23, 2020, following the public gathering where half a million bikers converged on Sturgis, August 7 through 16, 2020. Dave et al^23^ linked a deluge of cases to this event since social distancing and mask wearing were rare in Sturgis.^24,25^ Using anonymized cell phone data, they traced smartphone pings from nonresidents that indicated visitors came to Sturgis from all over the US, including states recently experiencing surges, making it a super-spreader event. Attendees were mostly adults, and more likely to come into contact with other adults rather than adolescents and youth, thereby increasing the number of cases in adults (including older adults). Indeed, a prior study^26^ we conducted on COVID-19 infections in South Dakota as of September 4, 2020, indicated the infection rate in 10-to-19-year-olds was 1.31 times that in adults 60 years and older (*P* < .001). Six weeks later, as cases from Sturgis developed, there was no longer a significant difference between the groups.

As for Pennsylvania and Michigan, political rallies, largely held without masking and social distancing, may have led to a reversal in pattern. In Pennsylvania, 15 political rallies were held from August to October 30. In Michigan, the data are from November 17, 2020, and in the months before the election, 11 political rallies were held there.^27^ These rallies attracted adults, including older adults, rather than adolescents and youth, and with the lack of social distancing and mask wearing,^28^ likely added to the caseloads. While there were political rallies in other states, they were far fewer: there were 6 in Florida (as of the date COVID-19 state data were tabulated, November 12, 2020), 1 in Oklahoma (October 22, 2020), 3 in Minnesota (October 23, 2020), and 6 in Wisconsin (October 23, 2020). The confluence of the Sturgis super-spreader event and political rallies likely inflated cases in older adults. There may have been other possible reasons for the differences in the 3 referenced states. Although the contribution of such political rallies is a potential explanation, further research should more thoroughly evaluate the temporal relationship of such rallies with increases in local COVID-19 infections.

Our finding of higher incidence in adolescents and youth for the wild-type strain paralleled the findings of the REACT-1 (Real-time Assessment of Community Transmission-1) UK study for B.1.617.2 (ie, the Delta variant), where there was a 5-fold higher positivity rate among individuals aged 5 to 12 years and those aged 18 to 24 years than among adults ages 65 years and older.^29^ These findings have been interpreted to mean that, for the Delta strain, youth drove the UK surge.^30^ However, our findings indicate that the high incidence in adolescents and youth was not novel and was present even with the wild-type strain. One reason the UK study found a 5-fold difference in positivity, which was greater than ours, may be that their study was conducted after vaccinations had been ongoing for many months for older adults in the UK. Our study was conducted prior to vaccine availability, and may be why the difference in incidence between the adolescents and youth vs older adults was less pronounced.

Our findings were also contrary to those of Dawood et al,^10^ but as noted earlier, that study had limitations. Dawood et al’s timeline conflated data for different variants, not all of which may have equal infectiousness for adolescents. Our findings show that for the original wild-type strain circulating in the US from the start of the pandemic through fall 2020, adolescents and youth had a significantly greater incidence of COVID-19 infection and greater relative risk of contracting COVID-19 than older adults.

### Limitations

This study had several limitations. One limitation noted earlier was sampling bias due to the undertesting of adolescents, which would lead to an underreporting of cases in this age bracket. Consequently, the incidence rates and relative risks of adolescents contracting COVID-19 were likely being underestimated in this study, and our finding that incidence rates of COVID-19 infection were greater in adolescents than in older adults was probably even stronger than indicated here. A second limitation was that the case data from the health department websites did not contain information on the sex of the participants in the various age brackets, and we were unable to know whether there were any corresponding differences in incidence rates. Another limitation was that having different durations for the different states precluded making comparisons between states.

## Conclusions

The findings of high incidence of COVID-19 in adolescents and youth in the US during fall 2020 were consistent with our earlier findings in Summer 2020. Our findings for the wild-type strain were also consistent with those of the REACT-1 study with the Delta strain.

The high incidence of COVID-19 cases among adolescents can inform decisions regarding vaccinations. For example, the Florida Department of Health recently issued guidance that Florida, contrary to US Centers for Disease Control and Prevention (CDC) recommendations, was formally recommending against COVID-19 vaccines for children ages 5 to 17 years.^31^ Recognition of the high adolescent infection rate and associated childhood mortality due to COVID-19 noted earlier—more than 1000 lives lost in the US in those under 18 years from the start of the pandemic through the beginning of March 2022—argues against Florida’s position and supports CDC recommendations.

## Notes

Supplement. **eFigure 1.** Proportion of Variants Circulating in the US from April 11, 2021, through April 24, 2021, Based on SARS-CoV-2 Genomes Sequenced During that Time Period **eAppendix 1.** Case Data by State Websites **eAppendix 2.** *New York Times* Websites Indicating Surges by State **eAppendix 3.** List of Supplemental Figures/Tables Containing Case Data **eAppendix 4.** Demographic Data by Age and by State Websites
