# Supplementary material for: Incidence and Relative Risk of COVID-19 in Adolescents and Youth Compared With Older Adults in 19 US States, Fall 2020
Source: JAMA Netw Open. 2022 Jul 15;5(7):e2222126. doi: 10.1001/jamanetworkopen.2022.22126 (PMC9287748; doi:10.1001/jamanetworkopen.2022.22126)
Supplement: Supplement. — eFigure 1. Proportion of Variants Circulating in the US from April 11, 2021, through April 24, 2021, Based on SARS-CoV-2 Genomes Sequenced During that Time Period eAppendix 1. Case Data by State Websites eAppendix 2. New York Times Websites Indicating Surges by State eAppendix 3. List of Supplemental Figures/Tables Containing Case Data eAppendix 4. Demographic Data by Age and by State Websites [file jamanetwopen-e2222126-s001.pdf]

# Supplemental Online Content

Schneiderman M, Rumain B, Kaganovskiy L, Geliebter A. Incidence and relative risk of COVID-19 in adolescents and youth compared with older adults in 19 US states, fall 2020. *JAMA Netw Open.* 2022;5(7):e2222126. doi:10.1001/jamanetworkopen.2022.22126

**eFigure 1.** Proportion of Variants Circulating in the US from April 11, 2021, through April 24, 2021, Based on SARS-CoV-2 Genomes Sequenced During that Time Period

**eAppendix 1.** Case Data by State Websites

**eAppendix 2.** *New York Times* Websites Indicating Surges by State

**eAppendix 3.** List of Supplemental Figures/Tables Containing Case Data

**eAppendix 4.** Demographic Data by Age and by State Websites

This supplemental material has been provided by the authors to give readers additional information about their work.

© 2022 Schneiderman M et al. *JAMA Network Open.*

## eFigure 1. Proportion of Variants Circulating in the US from April 11, 2021, through April 24, 2021, Based on SARS-CoV-2 Genomes Sequenced During that Time Period

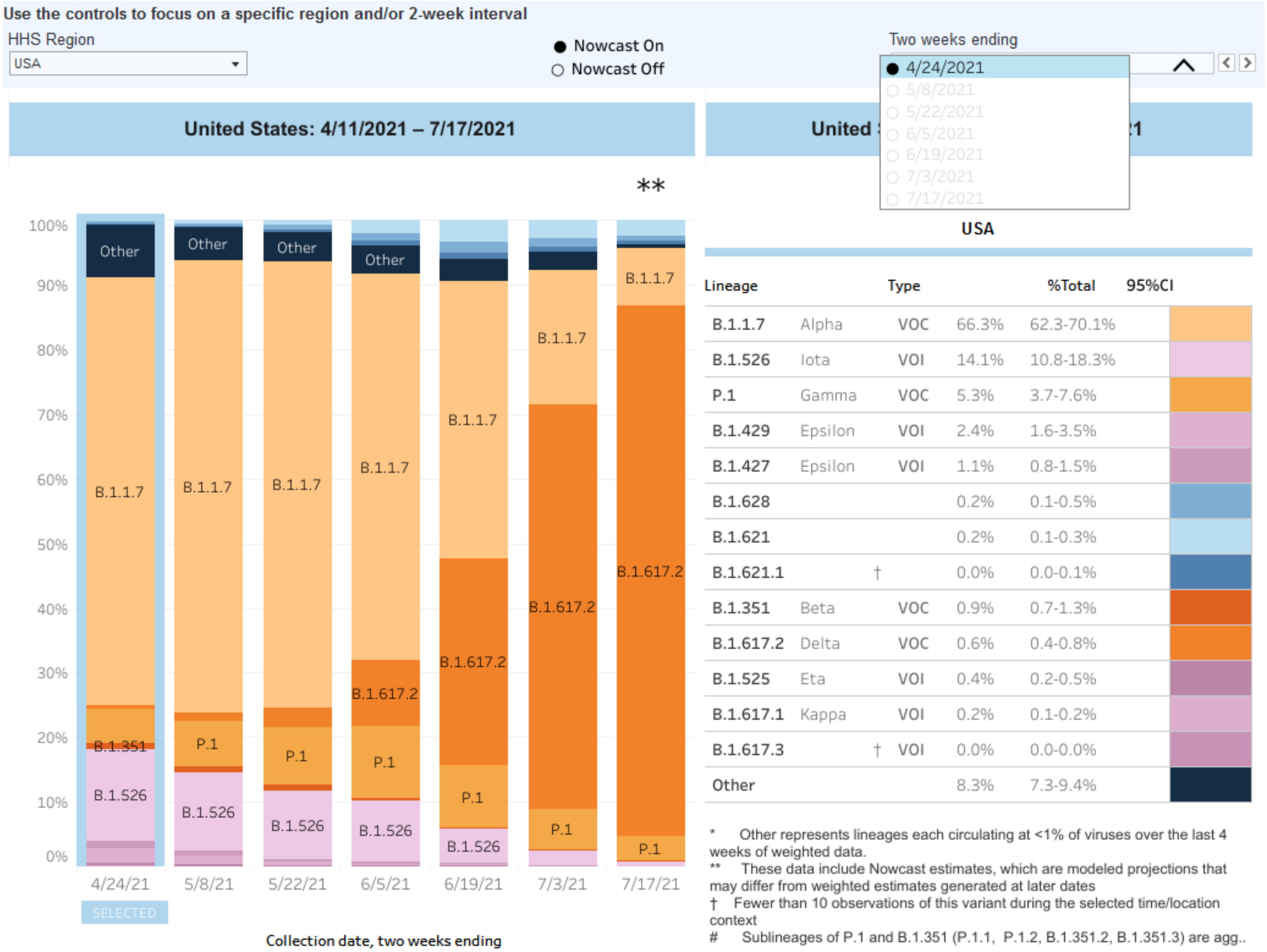


| Lineage | | Type | %Total | 95%CI |
|---|---|---|---|---|
| B.1.1.7 | Alpha | VOC | 66.3% | 62.3-70.1% |
| B.1.526 | Iota | VOI | 14.1% | 10.8-18.3% |
| P.1 | Gamma | VOC | 5.3% | 3.7-7.6% |
| B.1.429 | Epsilon | VOI | 2.4% | 1.6-3.5% |
| B.1.427 | Epsilon | VOI | 1.1% | 0.8-1.5% |
| B.1.628 | | | 0.2% | 0.1-0.5% |
| B.1.621 | | | 0.2% | 0.1-0.3% |
| B.1.621.1 | † | | 0.0% | 0.0-0.1% |
| B.1.351 | Beta | VOC | 0.9% | 0.7-1.3% |
| B.1.617.2 | Delta | VOC | 0.6% | 0.4-0.8% |
| B.1.525 | Eta | VOI | 0.4% | 0.2-0.5% |
| B.1.617.1 | Kappa | VOI | 0.2% | 0.1-0.2% |
| B.1.617.3 | † | VOI | 0.0% | 0.0-0.0% |
| Other | | | 8.3% | 7.3-9.4% |

* Other represents lineages each circulating at <1% of viruses over the last 4 weeks of weighted data.
** These data include Nowcast estimates, which are modeled projections that may differ from weighted estimates generated at later dates
† Fewer than 10 observations of this variant during the selected time/location context
# Sublineages of P.1 and B.1.351 (P.1.1, P.1.2, B.1.351.2, B.1.351.3) are agg..

Source: https://covid.cdc.gov/covid-data-tracker/#variant-proportions

© 2022 Schneiderman M et al. *JAMA Network Open.*

**eAppendix1.** Case Data by State Websites

(i)Alaska: https://experience.arcgis.com/experience/2d19dc2b5c7e4b399ff6495a8950493d/ [Retrieved Nov. 1, 2020]
(ii) Colorado: https://covid19.colorado.gov/data [Retrieved Oct. 23, 2020]
(iii) Michigan: https://www.mistartmap.info/compare/age-group [Retrieved Nov. 18, 2020]
(iv) Montana: https://dphhs.mt.gov/publichealth/cdepi/diseases/coronavirusmt/demographics [Retrieved Oct. 24, 2020]
(v) Nevada: https://nvhealthresponse.nv.gov [Retrieved Oct. 31, 2020]
(vi) New Mexico: https://cvprovider.nmhealth.org/public-dashboard.html# [Retrieved Oct. 24, 2020]
(vii) North Dakota: https://www.health.nd.gov/diseases-conditions/coronavirus/north-dakota-coronavirus-cases [Retrieved October 24, 2020]
(viii) Oregon: https://public.tableau.com/app/profile/oregon.health.authority.covid.19/viz/OregonCOVID-19CaseDemographicsandDiseaseSeverityStatewide/DemographicData [Retrieved Oct. 31, 2020]
(ix) Pennsylvania: https://experience.arcgis.com/experience/ed2def13f9b045eda9f7d22dbc9b500e [Retrieved Oct. 31, 2020]
(x) South Carolina: https://scdhec.gov/covid19/south-carolina-county-level-data-covid-19 [Retrieved May 15, 2021]
(xi) South Dakota: https://doh.sd.gov/news/coronavirus.aspx [Retrieved Oct. 24, 2020]
(xii) Tennessee: https://www.tn.gov/content/tn/health/cedep/ncov/data.html [Retrieved Nov. 12, 2020]
(xiii) Wisconsin: https://www.dhs.wisconsin.gov/covid-19/cases.htm#by%20age [Retrieved Oct. 24, 2020]
(xiv) Alabama: https://bamatracker.com/chart/caseages [Retrieved Nov. 14, 2020]
(xv) Florida: https://floridahealthcovid19.gov/ [Retrieved November 13, 2020]
(xvi)Oklahoma:https://coronavirus.health.ok.gov/sites/g/files/gmc786/f/2020.10.23_weekly_epi_report.pdf [Retrieved Oct. 23, 2020]
(xvii) Rhode Island: https://ri-department-of-health-covid-19-case-data-rihealth.hub.arcgis.com/#age [Retrieved October 25, 2020]
(xviii) Minnesota: https://www.health.state.mn.us/diseases/coronavirus/situation.html#ageg1[Retrieved Oct. 24, 2020]
(xix) Missouri: https://health.mo.gov/living/healthcondiseases/communicable/novel-coronavirus/data/public-health/demographics.php [Retrieved Oct. 24, 2020]

Since the data on the websites above are updated on a daily basis, we are including below, in the Supplemental Tables/Figures, the data as they appeared on the websites when we accessed them. These provide the numbers we used in our calculations.

© 2022 Schneiderman M et al. *JAMA Network Open.*

**eAppendix 2.** *New York Times* Websites Indicating Surges by State

https://www.nytimes.com/interactive/2021/us/alaska-covid-cases.html
https://www.nytimes.com/interactive/2021/us/colorado-covid-cases.html
https://www.nytimes.com/interactive/2021/us/michigan-covid-cases.html
https://www.nytimes.com/interactive/2021/us/montana-covid-cases.html
https://www.nytimes.com/interactive/2021/us/nevada-covid-cases.html
https://www.nytimes.com/interactive/2021/us/new-mexico-covid-cases.html
https://www.nytimes.com/interactive/2021/us/north-dakota-covid-cases.html
https://www.nytimes.com/interactive/2021/us/oregon-covid-cases.html
https://www.nytimes.com/interactive/2021/us/pennsylvania-covid-cases.html
https://www.nytimes.com/interactive/2021/us/south-carolina-covid-cases.html
https://www.nytimes.com/interactive/2020/us/south-dakota-coronavirus-cases.html
https://www.nytimes.com/interactive/2020/us/tennessee-coronavirus-cases.html
https://www.nytimes.com/interactive/2021/us/wisconsin-covid-cases.html
https://www.nytimes.com/interactive/2021/us/alabama-covid-cases.html
https://www.nytimes.com/interactive/2020/us/florida-coronavirus-cases.html
https://www.nytimes.com/interactive/2021/us/oklahoma-covid-cases.html
https://www.nytimes.com/interactive/2021/us/rhode-island-covid-cases.html
https://www.nytimes.com/interactive/2021/us/minnesota-covid-cases.html
https://www.nytimes.com/interactive/2020/us/missouri-coronavirus-cases.html

© 2022 Schneiderman M et al. *JAMA Network Open.*

**eAppendix 3.** List of Supplemental Figures/Tables Containing Case Data


1) eFigure A.  Alaska COVID-19 Cases by Age (as of October 31, 2020)
2) eTable B. Colorado COVID-19 Cases by Age (as of Oct. 22, 2020)
3) eFigure C. Michigan COVID-19 Cases by Age (as of Nov. 17, 2020)
4) eTable D. Montana COVID-19 Cases by Age (as of Oct. 23, 2020)
5) eFigure E.  Nevada COVID-19 Cases by Age (as of October 30, 2020)
6) eTable F. New Mexico COVID-19 Cases by Age (as of Oct. 23, 2020)
7) eTable G. North Dakota COVID-19 Cases by Age (as of October 23, 2020)
8) eTable/Figure H. Oregon COVID-19 Cases by Age (as of October 30. 2020)
9) eFigure I. Pennsylvania COVID-19 Cases by Age (as of October 30, 2020)
10) eFigure J. South Carolina COVID-19 Cases by Age (as of Dec. 5, 2020)
11) eFigure K.  South Dakota COVID-19 Cases by Age (as of Oct. 23, 2020)
12) eTable L. Tennessee COVID-19 Cases by Age (as of November 11, 2020)
13) eFigure M. Wisconsin COVID-19 Cases by Age (as of Oct. 23, 2020)
14) eTable/Figure N.  Alabama COVID-19 Cases by Age (as of November 13, 2020)
15) eTable/Figure O.  Florida COVID-19 Cases by Age (through November 12, 2020)
16) eTable/Figure P. Oklahoma COVID-19 Cases by Age (as of October 22, 2020)
17) eTable Q.   Rhode Island COVID-19 Cases by Age (as of October 24, 2020)
18) eTable R.  Minnesota COVID-19 Cases by Age (as of Oct. 23, 2020)
19) eTable S.  Missouri COVID-19 Cases by Age (as of Oct. 23, 2020)


© 2022 Schneiderman M et al. *JAMA Network Open.*

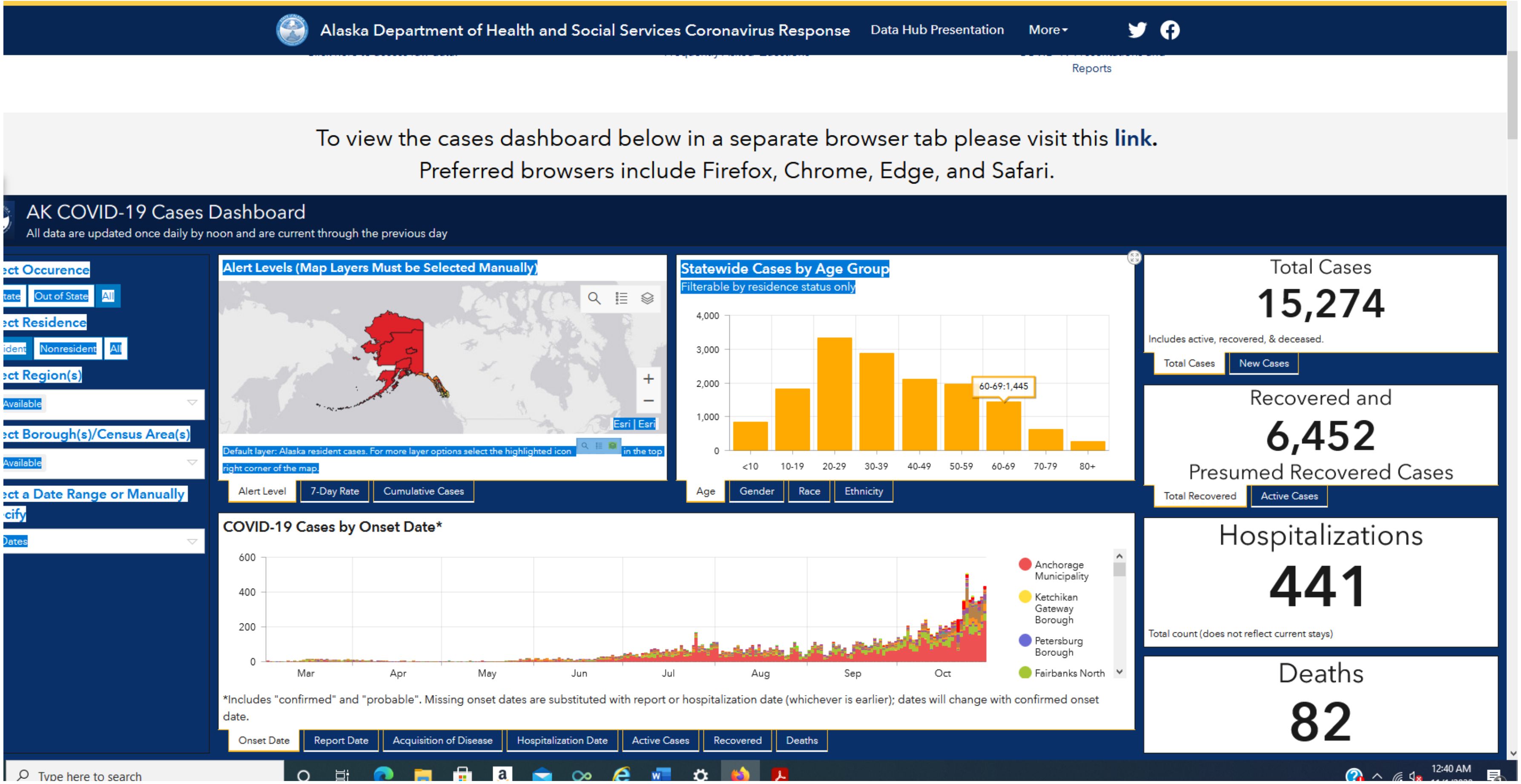


**eFigure A. Alaska COVID-19 Cases by Age (as of October 31, 2020)**

Source: Alaska Department of Health and Social Services Coronavirus Response Hub, AK COVID-19 Cases Dashboard
https://experience.arcgis.com/experience/2d19dc2b5c7e4b399ff6495a8950493d/

© 2022 Schneiderman M et al. *JAMA Network Open.*

## eTable B. Colorado COVID-19 Cases by Age (as of Oct. 22, 2020)

| Age | Proportion of Cases | Total Case Count |
|---|---|---|
| 0-9 | 0.038 | 3480 |
| 10-19 | 0.108 | 9890 |
| 20-29 | 0.223 | 20421 |
| 30-39 | 0.175 | 16025 |
| 40-49 | 0.154 | 14102 |
| 50-59 | 0.133 | 12179 |
| 60-69 | 0.085 | 7784 |
| 70-79 | 0.046 | 4212 |
| 80+ | 0.038 | 3480 |

Total cumulative cases: 91,572

Source: Colorado Department of Public Health and Environment, “CDPHE COVID19 State-Level Expanded Case Data” (as of 10/22/2020) https://covid19.colorado.gov/data

© 2022 Schneiderman M et al. *JAMA Network Open.*

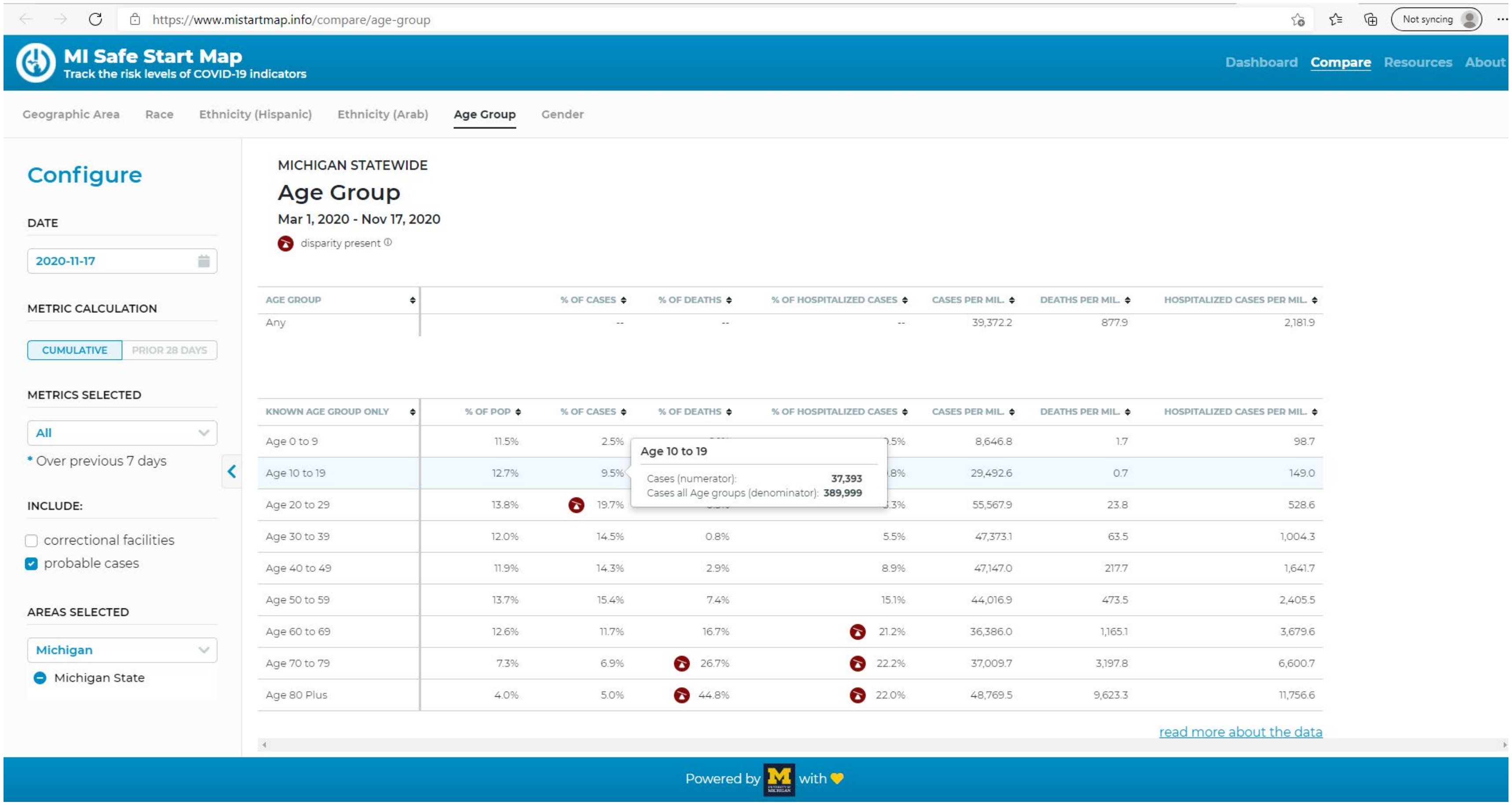


| AGE GROUP | % OF CASES | % OF DEATHS | % OF HOSPITALIZED CASES | CASES PER MIL. | DEATHS PER MIL. | HOSPITALIZED CASES PER MIL. |
|---|---|---|---|---|---|---|
| Any | -- | -- | -- | 39,372.2 | 877.9 | 2,181.9 |

| KNOWN AGE GROUP ONLY | % OF POP | % OF CASES | % OF DEATHS | % OF HOSPITALIZED CASES | CASES PER MIL. | DEATHS PER MIL. | HOSPITALIZED CASES PER MIL. |
|---|---|---|---|---|---|---|---|
| Age 0 to 9 | 11.5% | 2.5% | [illegible] | [illegible].5% | 8,646.8 | 1.7 | 98.7 |
| Age 10 to 19 | 12.7% | 9.5% | [illegible] | [illegible].8% | 29,492.6 | 0.7 | 149.0 |
| Age 20 to 29 | 13.8% | 19.7% | [illegible] | [illegible].3% | 55,567.9 | 23.8 | 528.6 |
| Age 30 to 39 | 12.0% | 14.5% | 0.8% | 5.5% | 47,373.1 | 63.5 | 1,004.3 |
| Age 40 to 49 | 11.9% | 14.3% | 2.9% | 8.9% | 47,147.0 | 217.7 | 1,641.7 |
| Age 50 to 59 | 13.7% | 15.4% | 7.4% | 15.1% | 44,016.9 | 473.5 | 2,405.5 |
| Age 60 to 69 | 12.6% | 11.7% | 16.7% | 21.2% | 36,386.0 | 1,165.1 | 3,679.6 |
| Age 70 to 79 | 7.3% | 6.9% | 26.7% | 22.2% | 37,009.7 | 3,197.8 | 6,600.7 |
| Age 80 Plus | 4.0% | 5.0% | 44.8% | 22.0% | 48,769.5 | 9,623.3 | 11,756.6 |

**eFigure C. Michigan COVID-19 Cases by Age (as of Nov. 17, 2020)**

Source: MI Safe Start Map, University of Michigan
https://www.mistartmap.info/compare/age-group

© 2022 Schneiderman M et al. *JAMA Network Open.*

**eTable D. Montana COVID-19 Cases by Age (as of Oct. 23, 2020)**

| Age Group | Number of Cases (percent of total) |
|---|---|
| 0-9 years | 1,148 (4%) |
| 10-19 years | 3,001 (11%) |
| 20-29 years | 5,452 (21%) |
| 30-39 years | 4,263 (16%) |
| 40-49 years | 3,514 (13%) |
| 50-59 years | 3,457 (13%) |
| 60-69 years | 2,883 (11%) |
| 70-79 years | 1,714 (6%) |
| 80-89 years | 796 (3%) |
| 90-99 years | 257 (1%) |
| 100+ | 18 (<1%) |
| Total Cases: | 26,503 |

Source: Montana Department of Public Health and Human Services
https://dphhs.mt.gov/publichealth/cdepi/diseases/coronavirusmt/demographics

© 2022 Schneiderman M et al. *JAMA Network Open.*

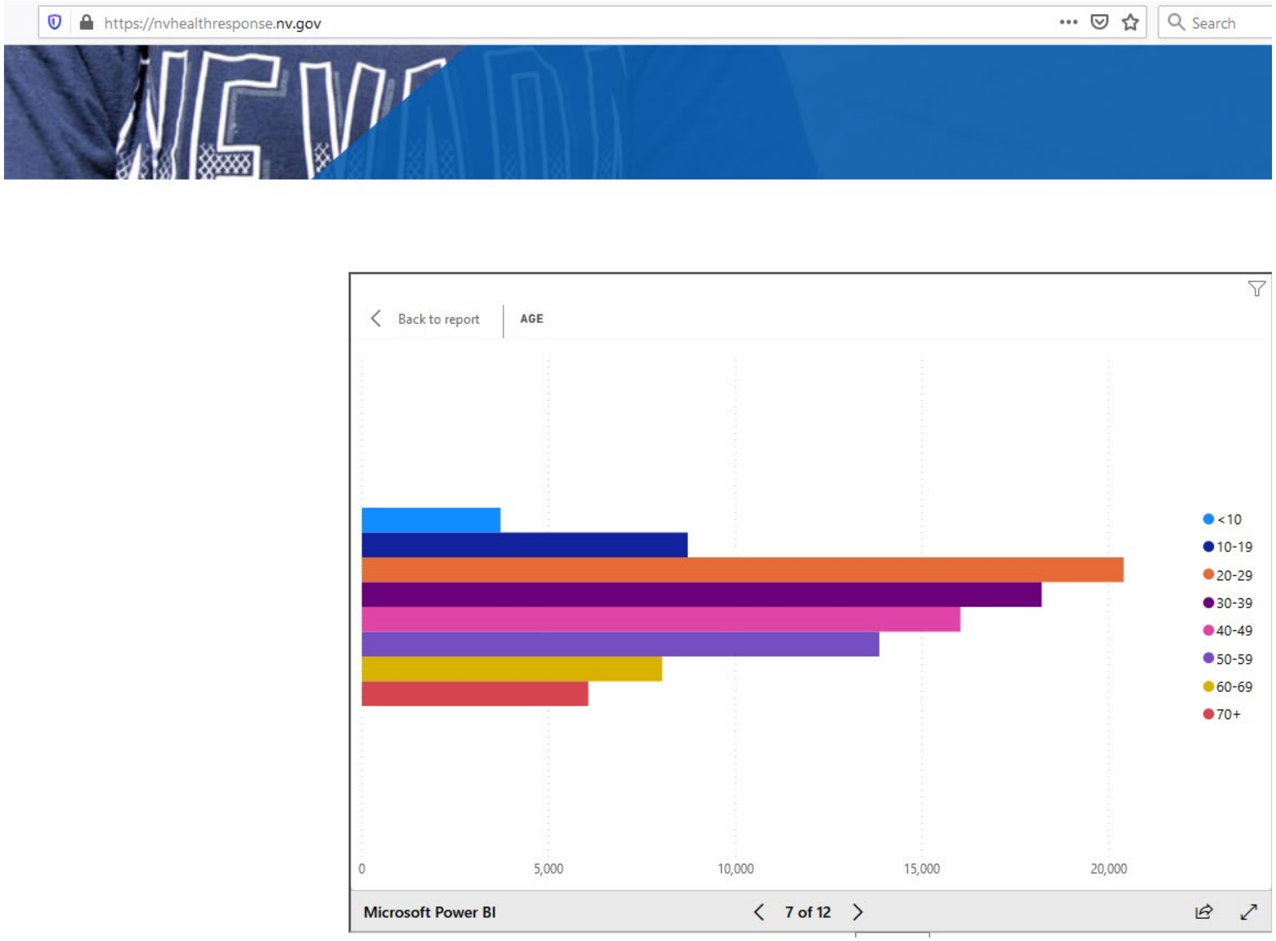


Total cases: 101,235

**eFigure E. Nevada COVID-19 Cases by Age (as of October 30, 2020)**

Source: Nevada Department of Health
https://nvhealthresponse.nv.gov

© 2022 Schneiderman M et al. *JAMA Network Open.*

## eTable F. New Mexico COVID-19 Cases by Age (as of 10/23/2020)

| Age | Number of Cases |
|---|---|
| 0-9 | 2120 |
| 10-19 | 4513 |
| 20-29 | 7987 |
| 30-39 | 7290 |
| 40-49 | 6145 |
| 50-59 | 5339 |
| 60-69 | 3594 |
| 70-79 | 1810 |
| 80-89 | 975 |
| 90+ | 347 |

Total: 4012

Source: New Mexico Department of Health
https://cvprovider.nmhealth.org/public-dashboard.html#

© 2022 Schneiderman M et al. *JAMA Network Open.*

## eTable G. North Dakota COVID-19 Cases by Age (as of October 23, 2020)

| Age | Total Cases |
|---|---|
| 0-9 | 1549 |
| 10-19 | 4658 |
| 20-29 | 8814 |
| 30-39 | 5932 |
| 40-49 | 4486 |
| 50-59 | 4292 |
| 60-69 | 3490 |
| 70-79 | 1859 |
| 80+ | 1794 |
| Total | 35,080 |

Source: North Dakota Department of Health
https://www.health.nd.gov/diseases-conditions/coronavirus/north-dakota-coronavirus-cases

© 2022 Schneiderman M et al. *JAMA Network Open.*

## eTable/Figure H. Oregon COVID-19 Cases by Age (as of October 30. 2020)

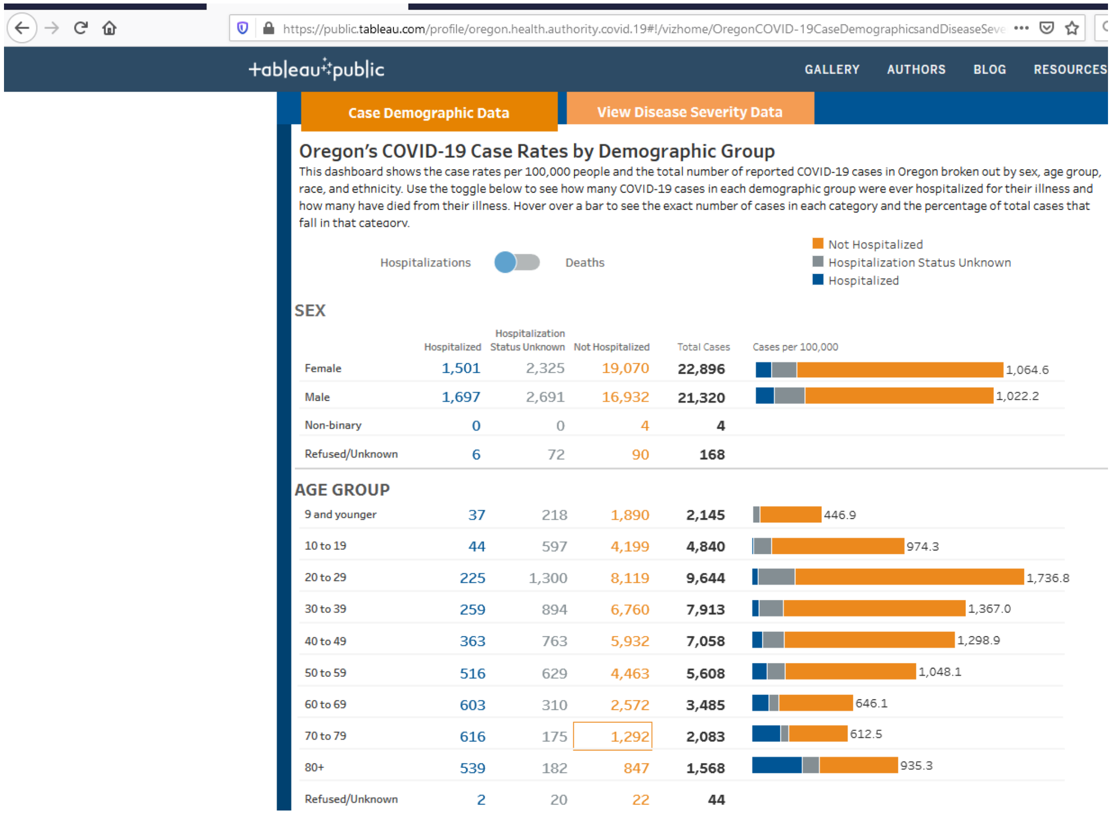

https://public.tableau.com/profile/oregon.health.authority.covid.19#!/vizhome/OregonCOVID-19CaseDemographicsandDiseaseSeve

+ableau+public GALLERY AUTHORS BLOG RESOURCES

Case Demographic Data | View Disease Severity Data

**Oregon's COVID-19 Case Rates by Demographic Group**

This dashboard shows the case rates per 100,000 people and the total number of reported COVID-19 cases in Oregon broken out by sex, age group, race, and ethnicity. Use the toggle below to see how many COVID-19 cases in each demographic group were ever hospitalized for their illness and how many have died from their illness. Hover over a bar to see the exact number of cases in each category and the percentage of total cases that fall in that category.

Hospitalizations / Deaths

Not Hospitalized; Hospitalization Status Unknown; Hospitalized

**SEX**

| | Hospitalized | Hospitalization Status Unknown | Not Hospitalized | Total Cases | Cases per 100,000 |
|---|---|---|---|---|---|
| Female | 1,501 | 2,325 | 19,070 | 22,896 | 1,064.6 |
| Male | 1,697 | 2,691 | 16,932 | 21,320 | 1,022.2 |
| Non-binary | 0 | 0 | 4 | 4 | |
| Refused/Unknown | 6 | 72 | 90 | 168 | |

**AGE GROUP**

| | Hospitalized | Hospitalization Status Unknown | Not Hospitalized | Total Cases | Cases per 100,000 |
|---|---|---|---|---|---|
| 9 and younger | 37 | 218 | 1,890 | 2,145 | 446.9 |
| 10 to 19 | 44 | 597 | 4,199 | 4,840 | 974.3 |
| 20 to 29 | 225 | 1,300 | 8,119 | 9,644 | 1,736.8 |
| 30 to 39 | 259 | 894 | 6,760 | 7,913 | 1,367.0 |
| 40 to 49 | 363 | 763 | 5,932 | 7,058 | 1,298.9 |
| 50 to 59 | 516 | 629 | 4,463 | 5,608 | 1,048.1 |
| 60 to 69 | 603 | 310 | 2,572 | 3,485 | 646.1 |
| 70 to 79 | 616 | 175 | 1,292 | 2,083 | 612.5 |
| 80+ | 539 | 182 | 847 | 1,568 | 935.3 |
| Refused/Unknown | 2 | 20 | 22 | 44 | |

Source: Oregon Health Authority (OHA) and the OHA Public Health Division
https://public.tableau.com/app/profile/oregon.health.authority.covid.19/viz/OregonCOVID-19CaseDemographicsandDiseaseSeverityStatewide/DemographicData

© 2022 Schneiderman M et al. *JAMA Network Open.*

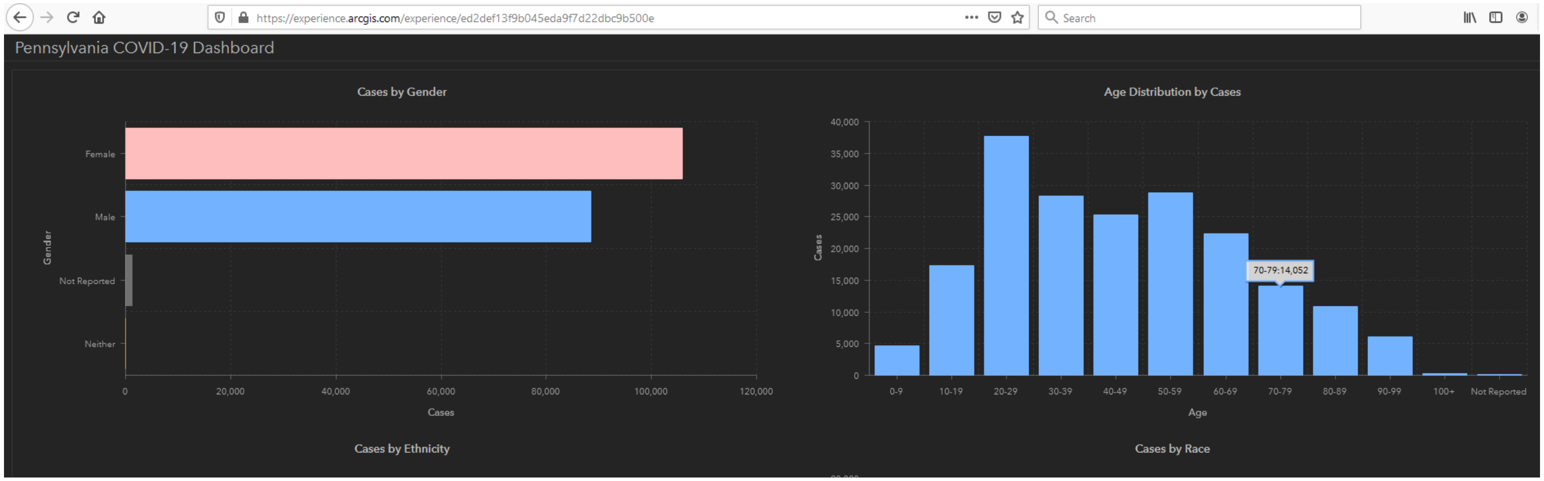


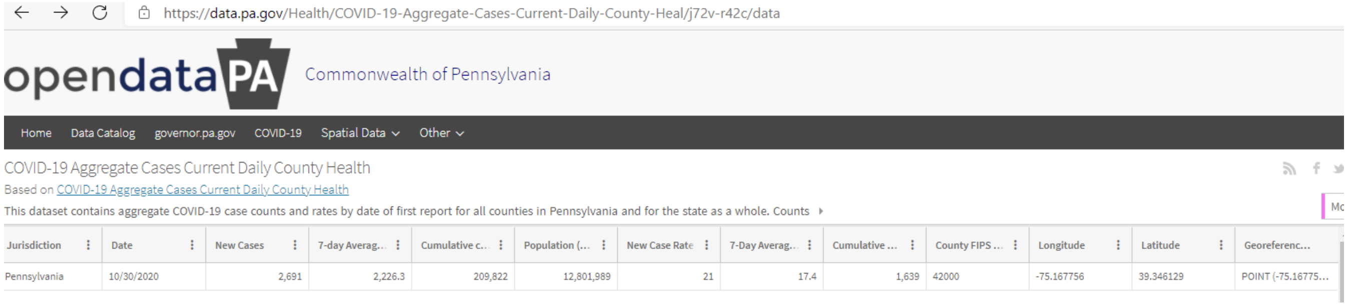


Total cases 209,822.

© 2022 Schneiderman M et al. *JAMA Network Open.*

**eFigure I. Pennsylvania COVID-19 Cases by Age (as of October 30, 2020)**

Source: Pennsylvania Department of Health, https://experience.arcgis.com/experience/ed2def13f9b045eda9f7d22dbc9b500e

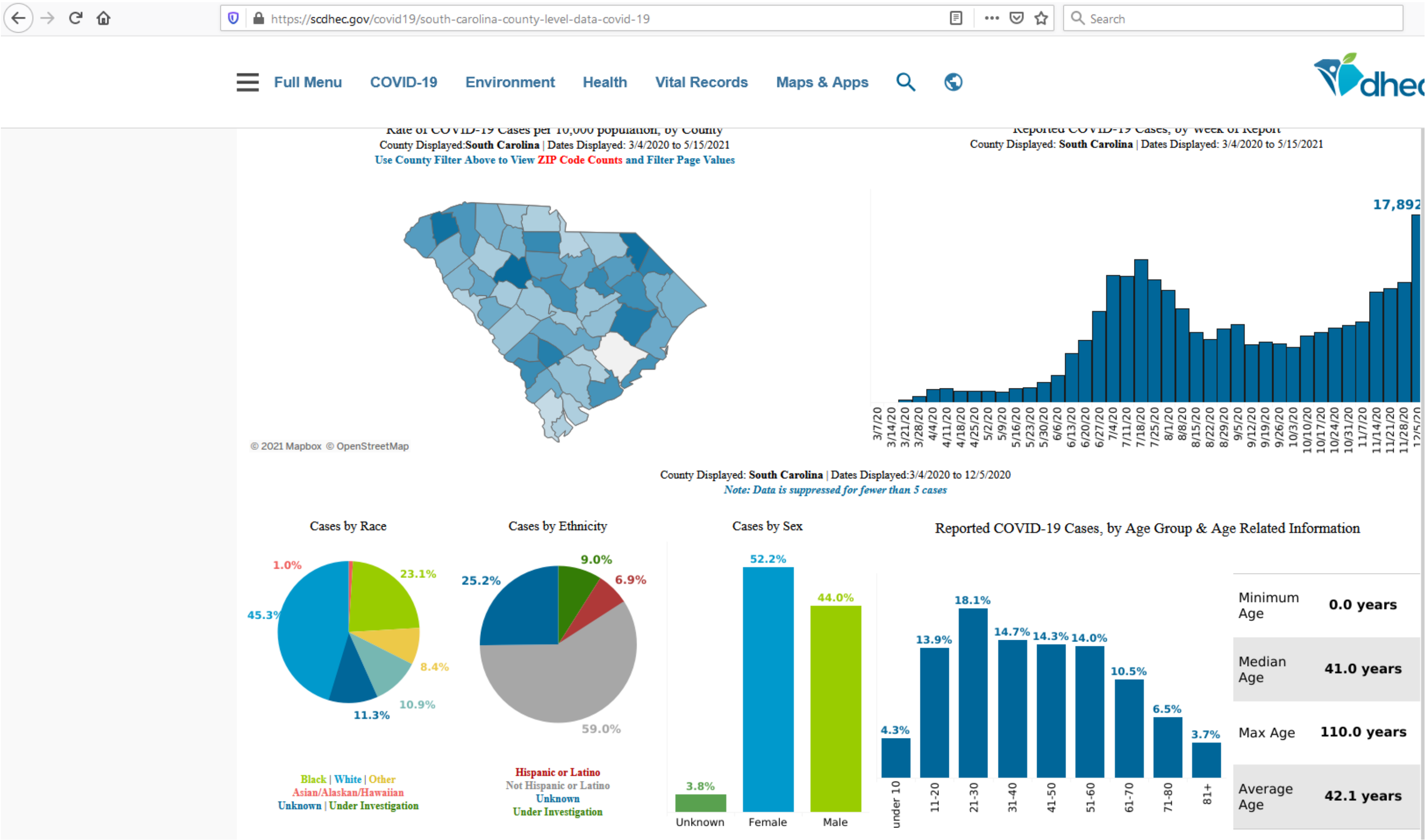


Total cases: 17,892

© 2022 Schneiderman M et al. *JAMA Network Open.*

**eFigure J. South Carolina COVID-19 Cases by Age (as of Dec. 5, 2020)**
Source: South Carolina Department of Health and Environmental Control
https://scdhec.gov/covid19/south-carolina-county-level-data-covid-19

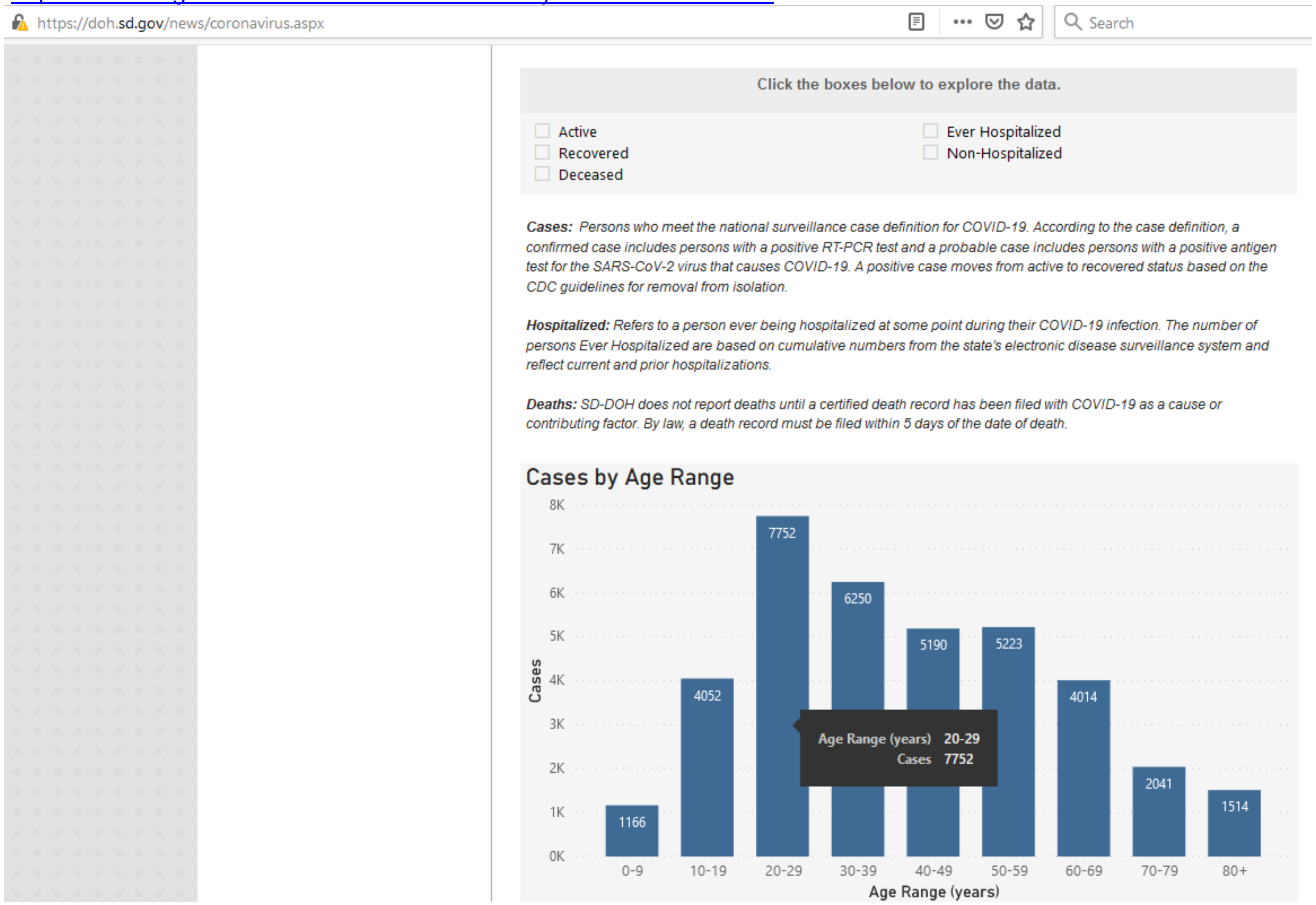


Total cases: 37,262

© 2022 Schneiderman M et al. *JAMA Network Open.*

**eFigure K. South Dakota COVID-19 Cases by Age (as of Oct. 23, 2020)**
Source: South Dakota Department of health
https://doh.sd.gov/news/coronavirus.aspx

**eTable L. Tennessee COVID-19 Cases by Age (as of November 11, 2020)**

| Age | Total Cases |
|---|---|
| 0-10 | 14,363 |
| 11-20 | 38,925 |
| 21-30 | 57,829 |
| 31-40 | 46,794 |
| 41-50 | 43,426 |
| 51-60 | 39,243 |
| 61-70 | 27,287 |
| 71-80 | 16,316 |
| 81+ | 8,801 |
| Pending | 397 |
| Total | 293,381 |

Source: Tennessee Department of Health, "Age Dataset" in https://www.tn.gov/health/cedep/ncov/data/downloadable-datasets.html

© 2022 Schneiderman M et al. *JAMA Network Open.*

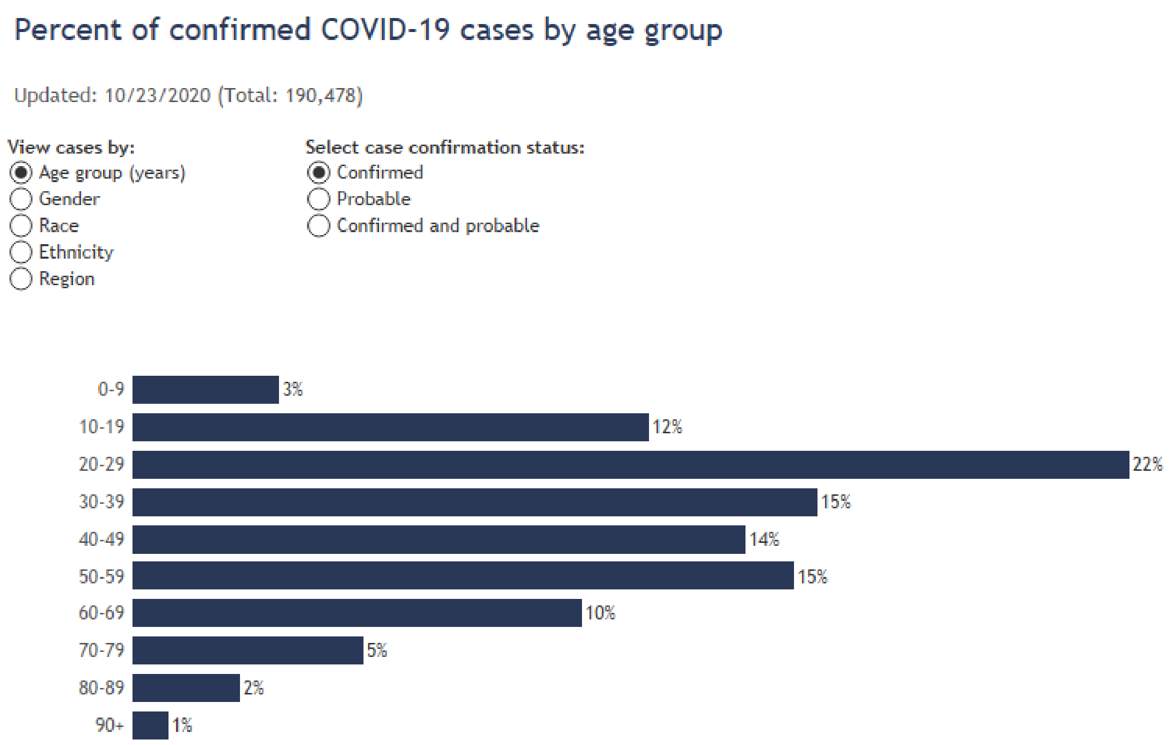


**eFigure M. Wisconsin COVID-19 Cases by Age (as of Oct. 23, 2020)**

Source: Wisconsin Department of Health Services
https://www.dhs.wisconsin.gov/covid-19/cases.htm#by%20age

© 2022 Schneiderman M et al. *JAMA Network Open.*

## eTable/Figure N. Alabama COVID-19 Cases by Age (as of November 13, 2020)

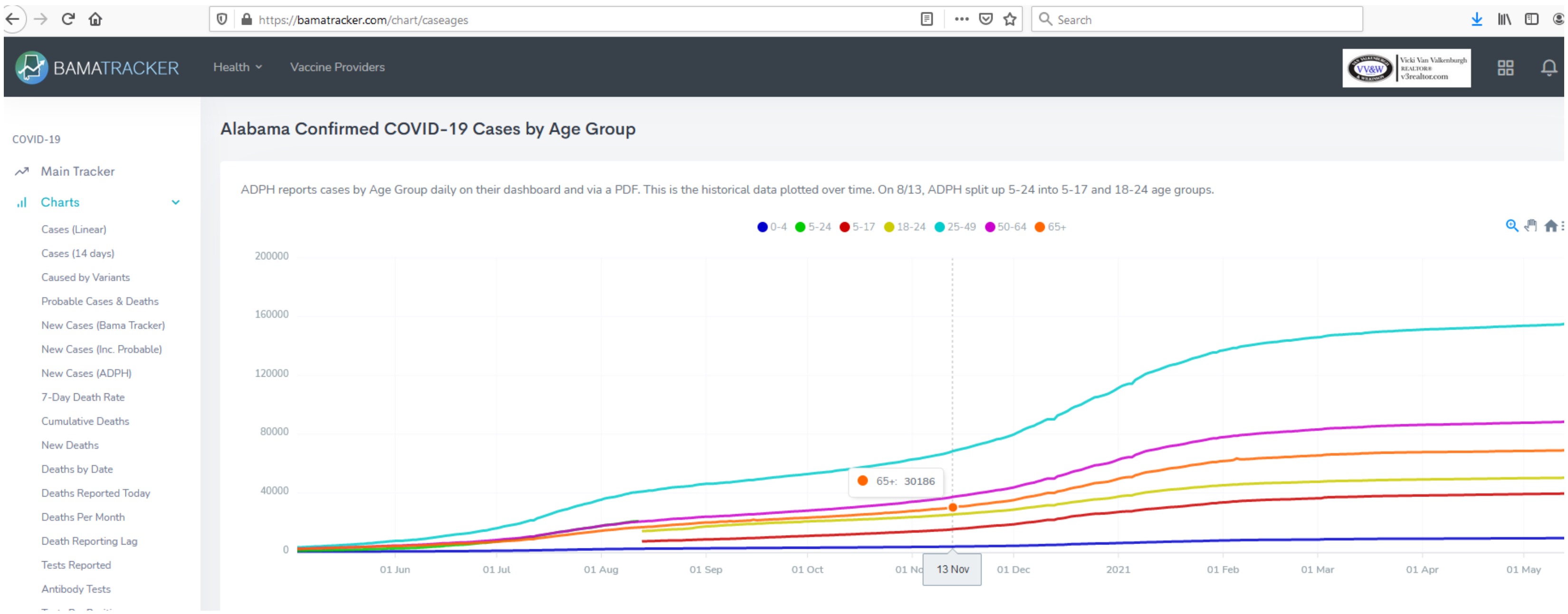


| Age | Cumulative Cases |
|---|---|
| 0-4 | 3,519 |
| 5-17 | 15,316 |
| 18-24 | 25,413 |
| 25-49 | 68,497 |
| 50-64 | 37,344 |
| 65+ | 30,186 |

Source: Alabama Department of Public Health COVID-19 Dashboard Hub
https://bamatracker.com/chart/caseages

© 2022 Schneiderman M et al. *JAMA Network Open.*

**eTable/Figure O. Florida COVID-19 Cases by Age (through November 12, 2020)**

# COVID-19: characteristics of 858,585 Florida resident cases

**Data through Nov 12, 2020 verified as of Nov 13, 2020 at 09:25 AM**

Data in this report are provisional and subject to change.

| Age group | Cases | | Hospitalizations | | Deaths | |
|---|---|---|---|---|---|---|
| 0-4 years | 14,429 | 2% | 336 | 1% | 0 | 0% |
| 5-14 years | 39,056 | 5% | 310 | 1% | 5 | 0% |
| 15-24 years | 140,515 | 16% | 1,376 | 3% | 32 | 0% |
| 25-34 years | 156,421 | 18% | 2,870 | 6% | 101 | 1% |
| 35-44 years | 135,609 | 16% | 4,282 | 8% | 302 | 2% |
| 45-54 years | 133,416 | 16% | 6,404 | 12% | 714 | 4% |
| 55-64 years | 111,472 | 13% | 9,001 | 17% | 1,920 | 11% |
| 65-74 years | 67,147 | 8% | 10,310 | 20% | 3,726 | 21% |
| 75-84 years | 38,283 | 4% | 9,751 | 19% | 5,096 | 29% |
| 85+ years | 21,217 | 2% | 6,895 | 13% | 5,549 | 32% |
| Unknown | 1,020 | 0% | 7 | 0% | 0 | 0% |
| **Total** | **858,585** | | **51,542** | | **17,445** | |

| Gender | Ca[illegible] |
|---|---|
| Female | 441,75[illegible] |
| Male | 412,38[illegible] |
| Unknown | 4,44[illegible] |
| **Total** | **858,58[illegible]** |

Source: Florida Department of Health, https://floridahealthcovid19.gov/

© 2022 Schneiderman M et al. *JAMA Network Open.*

## eTable/Figure P. Oklahoma COVID-19 Cases by Age (as of Oct. 22, 2020)

**DEMOGRAPHIC INFORMATION as of October 22, 2020**

| | Cases count (%)[1] | | Deaths count (%)[1] | | Cumulative Incidence Rate[2] | Cumulative Mortality Rate[2] |
|---|---|---|---|---|---|---|
| **Oklahoma** | 112,483 | | 1221 | | 2,852.7 | 31.0 |
| **Gender** | | | | | | |
| Male | 53,835 | (47.9) | 680 | (55.7) | 2,756.5 | 34.8 |
| Female | 58,648 | (52.1) | 541 | (44.3) | 2,947.1 | 27.2 |
| **Age group** | | | | | | |
| Under 1- 4 | 2,126 | (1.9) | 0 | (0.0) | 816.3 | 0.0 |
| 5-14 | 6,392 | (5.7) | 1 | (0.1) | 1,188.3 | 0.2 |
| 15-24 | 22,499 | (20.0) | 2 | (0.2) | 4,183.3 | 0.4 |
| 25-34 | 19,221 | (17.1) | 11 | (0.9) | 3,538.4 | 2.0 |
| 35-44 | 17,855 | (15.9) | 19 | (1.6) | 3,641.0 | 3.9 |
| 45-54 | 15,456 | (13.7) | 56 | (4.6) | 3,357.7 | 12.2 |
| 55-64 | 13,080 | (11.6) | 150 | (12.3) | 2,650.8 | 30.4 |
| 65-74 | 8,698 | (7.7) | 324 | (26.5) | 2,425.2 | 90.3 |
| 75-84 | 4,771 | (4.2) | 333 | (27.3) | 2,541.7 | 177.4 |
| 85+ | 2,384 | (2.1) | 325 | (26.6) | 3,257.0 | 444.0 |
| Unknown | 1 | (0.0) | | | | |

Source: Oklahoma State Department of Health
https://coronavirus.health.ok.gov/sites/g/files/gmc786/f/2020.10.23_weekly_epi_report.pdf

© 2022 Schneiderman M et al. *JAMA Network Open.*

## eTable Q. Rhode Island COVID-19 Cases by Age (as of October 24, 2020)

| Age | Cumulative Cases |
|---|---|
| 0-4 | 480 |
| 5-9 | 531 |
| 10-14 | 656 |
| 15-18 | 1174 |
| 19-24 | 3522 |
| 25-29 | 2655 |
| 30-39 | 4658 |
| 40-49 | 4125 |
| 50-59 | 4175 |
| 60-69 | 2880 |
| 70-79 | 1773 |
| 80+ | 2364 |
| Total cases | 28,993 |

Source: Rhode Island Department of Health (RIDOH) SalesForce COVID-19 Case Dataset
https://ri-department-of-health-covid-19-case-data-rihealth.hub.arcgis.com/#age

© 2022 Schneiderman M et al. *JAMA Network Open.*

## eTable R. Minnesota COVID-19 Cases by Age (as of Oct. 23, 2020)

| Age Group | Number of Cases |
|---|---|
| **0-4 years** | 2,536 |
| **5-9 years** | 2,624 |
| **10-14 years** | 3,988 |
| **15-19 years** | 11,769 |
| **20-24 years** | 16,429 |
| **25-29 years** | 12,611 |
| **30-34 years** | 11,466 |
| **35-39 years** | 10,447 |
| **40-44 years** | 9,371 |
| **45-49 years** | 8,916 |
| **50-54 years** | 8,893 |
| **55-59 years** | 8,242 |
| **60-64 years** | 6,536 |
| **65-69 years** | 4,415 |
| **70-74 years** | 3,393 |
| **75-79 years** | 2,561 |
| **80-84 years** | 2,066 |
| **85-89 years** | 1,754 |
| **90-94 years** | 1,209 |
| **95-99 years** | 524 |
| **100+ years** | 95 |
| **Unknown/missing** | 18 |
| Total | 129,863 |

Source: https://www.health.state.mn.us/diseases/coronavirus/situation.html#ageg1

© 2022 Schneiderman M et al. *JAMA Network Open.*

**eTable S. Missouri COVID-19 Cases by Age (as of Oct. 23, 2020)**

| Age | Missouri Number of Cases |
|---|---|
| 0-9 | 3054 |
| 10-19 | 13637 |
| 20 | 3596 |
| 21 | 3512 |
| 22 | 2831 |
| 23 | 2655 |
| 24 | 2403 |
| 25 | 2316 |
| 26 | 2260 |
| 27 | 2243 |
| 28 | 2181 |
| 29 | 2114 |
| 30 | 2006 |
| 31 | 1942 |
| 32 | 1893 |
| 33 | 1762 |
| 34 | 1769 |
| 35 | 1761 |
| 36 | 1659 |
| 37 | 1761 |
| 38 | 1736 |
| 39 | 1761 |
| 40 | 1752 |
| 41 | 1554 |
| 42 | 1589 |
| 43 | 1659 |
| 44 | 1579 |
| 45 | 1654 |
| 46 | 1556 |
| 47 | 1620 |
| 48 | 1678 |
| 49 | 1783 |
| 50 | 1686 |
| 51 | 1704 |
| 52 | 1616 |

© 2022 Schneiderman M et al. *JAMA Network Open.*

| | |
|---|---|
| 53 | 1619 |
| 54 | 1579 |
| 55 | 1655 |
| 56 | 1633 |
| 57 | 1677 |
| 58 | 1619 |
| 59 | 1608 |
| 60 | 1519 |
| 61 | 1477 |
| 62 | 1361 |
| 63 | 1394 |
| 64 | 1308 |
| 65 | 1188 |
| 66 | 1100 |
| 67 | 1028 |
| 68 | 1031 |
| 69 | 976 |
| 70 | 950 |
| 71 | 886 |
| 72 | 908 |
| 73 | 844 |
| 74 | 731 |
| 75 | 707 |
| 76 | 679 |
| 77 | 746 |
| 78 | 627 |
| 79 | 574 |
| 80-84 | 2410 |
| 85-89 | 1918 |
| 90+ | 1975 |
| Unknown | 289 |

Total cases: 120,298

Source: Missouri Department of Health and Senior Services
https://health.mo.gov/living/healthcondiseases/communicable/novel-coronavirus/cases-by-age.php

© 2022 Schneiderman M et al. *JAMA Network Open.*

**eAppendix 4.** Demographic Data by Age and by State Websites

(i) Alaska: https://live.laborstats.alaska.gov/pop/
(ii) Colorado: Colorado State Demography Office, Colorado Department of Local Affairs, https://demography.dola.colorado.gov/population/data/sya-regions/
(iii) Michigan: https://censusreporter.org/profiles/04000US26-michigan/
(iv) Montana: https://censusreporter.org/profiles/04000US30-montana/
(v) Nevada: https://censusreporter.org/profiles/04000US32-nevada/
(vi) New Mexico: https://censusreporter.org/data/table/?table=B01001&geo_ids=04000US35&primary_geo_id=04000US35
(vii) North Dakota: https://censusreporter.org/profiles/04000US38-north-dakota/
(viii) Oregon: https://censusreporter.org/profiles/04000US41-oregon/
(ix) Pennsylvania: https://censusreporter.org/profiles/04000US42-pennsylvania/
(x) South Carolina https://censusreporter.org/profiles/04000US45-south-carolina/
(xi) South Dakota: https://www.sdstate.edu/sociology-rural-studies/census-data-center/population-change
(xii) Tennessee: https://www.tn.gov/content/dam/tn/health/documents/population/TN-Population-by-AgeGrp-Sex-Race-Ethnicity-2019.pdf
(xiii) Wisconsin: https://censusreporter.org/profiles/04000US55-wisconsin/
(xiv) Alabama: https://censusreporter.org/profiles/04000US01-alabama/
(xv) Florida: http://edr.state.fl.us/Content/populationdemographics/data/Pop_Census_Day.pdf
(xvi) Oklahoma: https://censusreporter.org/profiles/04000US40-oklahoma/
(xvii) Rhode Island: https://censusreporter.org/profiles/04000US44-rhode-island/
(xviii) Minnesota: https://censusreporter.org/profiles/04000US27-minnesota/
(xix) Missouri: https://healthapps.dhss.mo.gov/MoPhims/QueryBuilder?qbc=PNM&q=1&m=1

© 2022 Schneiderman M et al. *JAMA Network Open.*
